# Lipid Profile in Primary Aldosteronism: Cross‐Sectional and Post‐Treatment Analyses

**DOI:** 10.1002/lipd.70051

**Published:** 2026-03-17

**Authors:** Meriem Yazidi, Arige Abid, Chayma Bel hadj Sliman, Elyes Kamoun, Ibtissem Oueslati, Fatma Chaker, Nadia Khessairi, Melika Chihaoui

**Affiliations:** ^1^ Department of Endocrinology University of Tunis el Manar, Faculty of Medicine of Tunis, La Rabta Hospital Tunis Tunisia

**Keywords:** adrenalectomy, dyslipidemia, essential hypertension, mineralocorticoid receptor antagonists, primary aldosteronism, renal function, triglycerides

## Abstract

The relationship between primary aldosteronism (PA) and lipid metabolism remains controversial, with inconsistent findings reported in the literature. This study aimed to clarify PA's impact on the lipid profile using both a cross‐sectional comparison with essential hypertension (EH) controls and a longitudinal within‐patient pre‐post treatment analysis in a North African tertiary‐center cohort. This retrospective study included 112 patients with PA and 115 contemporaneously hospitalized patients with EH in whom secondary hypertension had been excluded. Sociodemographic, clinical, and biochemical variables (fasting blood glucose, HbA1c, lipid profile, creatinine, potassium, baseline aldosterone levels, and aldosterone‐to‐renin ratio), as well as therapeutic data, were collected at admission in both groups. In the PA group, clinical and biological parameters performed 1 year after specific PA management were recorded. Triglyceride levels were significantly higher in patients with PA not receiving lipid‐lowering therapy (1.47 (1.13–1.92) vs. 1.24 (1.02–1.69) mmol/L; *p* = 0.04) but this difference disappeared after multivariable adjustment for age, sex, BMI, and fasting glucose. A baseline aldosterone level > 200 pg/mL was associated with lower triglyceride levels (1.35 (1.02–1.81) vs. 1.81 (1.35–2.15) mmol/L; *p* = 0.01). Patients with PA who underwent adrenalectomy demonstrated a significant increase in triglyceride levels at 1 year (1.52 (1.32–2.11) vs. 2.08 (1.38–2.50) mmol/L; *p* = 0.04). The estimated glomerular filtration rate declined significantly 1 year after PA specific treatment (84.9 ± 19.7 vs. 73.6 ± 24.9 mL/min/1.73 m^2^; *p* < 0.01). Our findings show mixed associations between aldosterone and triglyceride levels, which require confirmation in prospective studies.

## 1 Introduction

Primary aldosteronism (PA) is characterized by autonomous and excessive secretion of aldosterone by the adrenal glands, most commonly due to unilateral adenoma or bilateral adrenal hyperplasia. PA is now established as the leading cause of secondary hypertension, accounting for up to 10% of hypertension (Funder 2016). It is associated with significantly increased cardiovascular risk, with higher rates of major cardiovascular events compared to essential hypertension (EH) (Turchi et al. 2014; Monticone et al. 2018). Aldosterone exerts direct deleterious effects on the cardiovascular system, promoting myocardial fibrosis, left ventricular hypertrophy, and vascular remodeling, and further contributes to inflammatory and prothrombotic changes that exacerbate this risk (Turchi et al. 2014; Stehr et al. 2010). Beyond hypertension, emerging evidence suggests that PA may also influence lipid metabolism. However, findings remain inconsistent. While several studies report no association (Sun et al. 2024; Huang et al. 2021; Tsurutani et al. 2017; Matrozova et al. 2009), others describe a more adverse lipid profile in patients with PA (Bochud et al. 2006; Hannich et al. 2018; Jochmanová et al. 2013; Musani et al. 2013). Conversely, some data suggest a potentially beneficial impact on lipid metabolism (Zhang et al. 2020; Adolf et al. 2016; Moon et al. 2021; Manosroi et al. 2022; Kaga et al. 2015). Characterizing the lipid profile of patients with PA is therefore essential for comprehensive cardiovascular risk assessment and for guiding preventive and therapeutic strategies. Moreover, most available data are limited to single time‐point comparisons, while longitudinal within‐patient changes after specific PA treatment have been less frequently investigated. In addition, evidence from North African and Middle Eastern populations remains scarce, although cardiometabolic risk profiles may differ across ethnic and regional backgrounds. In this context, we aimed to (i) compare the lipid profiles of Tunisian patients with PA and those with EH, (ii) assess the relationship between lipid parameters and the biological severity of PA, and (iii) evaluate changes in lipid parameters following specific treatment of PA.

## 2 Methods

Data were extracted from the medical records of patients hospitalized in the Endocrinology Department of La Rabta University Hospital (Tunis, Tunisia) between January 1, 2001, and December 31, 2023. The reporting of this study follows the RECORD (Reporting of studies Conducted using Observational Routinely collected Data) statement. Only patients with complete medical records regarding the primary endpoints (lipid profile and PA diagnostic work‐up) were included in the final analysis. A total of 112 patients with PA and 115 with EH were included. Patients with PA had either a baseline aldosterone level > 200 pg/mL associated with undetectable renin and hypokalemia (< 3.5 mmol/L), or an aldosterone‐to‐renin ratio (ARR) > 23 (with both aldosterone and plasma renin expressed in pg/mL) associated with a positive confirmatory test. The confirmatory tests used in our department were either the captopril challenge test or the saline infusion test. Controls were hypertensive patients hospitalized during the same period who had a negative etiological work‐up for secondary hypertension, defined as follows: Serum cortisol levels < 50 nmol/L (1.8 μg/dL) after a 1 mg dexamethasone suppression test, or normal urinary free cortisol; normal urinary or plasma metanephrines; normal baseline aldosterone (< 150 pg/mL) and ARR (< 23); normal renal arteries on Doppler ultrasound or computed tomography angiography; and no evidence of renal or secondary causes of hypertension.

Exclusion criteria for both patients and controls included conditions known to affect lipid metabolism, such as chronic inflammatory, infectious, or neoplastic diseases; severe renal impairment (Glomerular filtration rate < 30 mL/min/1.73 m^2^); hepatic failure; endocrine disorders associated with metabolic disturbances (e.g., thyroid dysfunction, polycystic ovary syndrome, hypogonadism, hypopituitarism); long‐term corticosteroids or estrogen–progestin therapy; pregnancy or lactation; and cases in which the conditions for aldosterone and renin measurement did not comply with the 2016 Endocrine Society Clinical Practice Guidelines (Bornstein et al. 2016).

Sociodemographic, clinical, biological, and therapeutic data were collected at admission. Smoking status, history of cardiovascular events, diabetes, and dyslipidemia, along with current treatments, were documented for both groups. Weight, height, waist circumference (WC), and systolic and diastolic blood pressures were recorded. Body mass index (BMI) was calculated as weight divided by height squared (kg/m^2^). Blood pressure was measured by medical staff using calibrated sphygmomanometers during hospitalization. Measurements were taken in a seated position after at least 5 min of rest. Blood pressure values were recorded to the nearest 5 mmHg, which explains the distribution of median values.

Routine biochemical assays were performed at the Biochemistry Department of La Rabta University Hospital. Hormonal measurements (plasma aldosterone and renin) were centralized at the Institut Pasteur de Tunis throughout the study period. Although assay kits and calibration standards evolved over time in accordance with laboratory practice, all measurements were performed using radioimmunological techniques (RIA/IRMA) within the same reference laboratory, with internal quality controls and period‐specific reference ranges applied. The following biological parameters were recorded for subjects in both groups: baseline aldosterone, ARR, fasting blood glucose, HbA1c in subjects with diabetes, total cholesterol, triglycerides, HDL cholesterol, creatinine and potassium levels. The lipid profile and fasting blood glucose are collected after at least 12 h of fasting in our department. LDL cholesterol was calculated using the Friedewald formula when triglyceride levels were below 4.5 mmol/L (Martins et al. 2022). When triglyceride levels were ≥ 4.5 mmol/L, LDL cholesterol was not calculated and was treated as missing data, as direct LDL measurements were not routinely available; this concerned fewer than five patients and therefore resulted in negligible missing data. The glomerular filtration rate (eGFR) was determined using the Modification of Diet in Renal Disease (MDRD) equation (Levey et al. 2006).

All patients with PA underwent adrenal CT imaging as part of routine evaluation. Adrenal venous sampling was not available; therefore, no etiological subtype classification (unilateral vs. bilateral disease) was attempted, and imaging findings were reported descriptively only.

In patients with PA, treatment (adrenalectomy or mineralocorticoid receptor antagonists) was documented. BMI, systolic and diastolic blood pressures, lipid parameters, eGFR, and potassium levels performed 1 year after specific PA treatment were recorded. Follow‐up data were collected within a window of 12 ± 3 months after PA treatment to ensure clinical stability while minimizing the confounding effects of aging or long‐term therapeutic modifications.

The study was approved by the ethics committee of the La Rabta Hospital (CERB 61/2025).

### 2.1 Statistical Analysis

Data were analyzed using SPSS version 23.0 (IBM Corp., Armonk, NY, USA). Normality of quantitative variables was tested with the Kolmogorov–Smirnov test. Qualitative variables were expressed as counts and percentages; quantitative variables as mean ± SD or median (IQR), as appropriate. Between‐group comparisons were performed using the Student's *t*‐test or Mann–Whitney test for quantitative variables, and the Chi‐square or Fisher's exact test for categorical variables. Correlations between quantitative variables were assessed with Spearman's test. Within‐group comparisons before and after PA treatment were performed using the paired Student's *t*‐test or Wilcoxon signed‐rank test for quantitative variables, and McNemar's test for categorical variables. In comparing lipid parameters between the PA and EH groups, multivariable linear regression analysis was used to adjust for factors known to influence the lipid profile. Two models were specified: Model 1 adjusted for age and sex (total association), and Model 2 further adjusted for BMI and fasting plasma glucose, considered potential metabolic mediators, to assess the direct association independent of body composition and insulin resistance. Statistical significance was defined as *p* < 0.05.

## 3 Results

**FIGURE 1:**
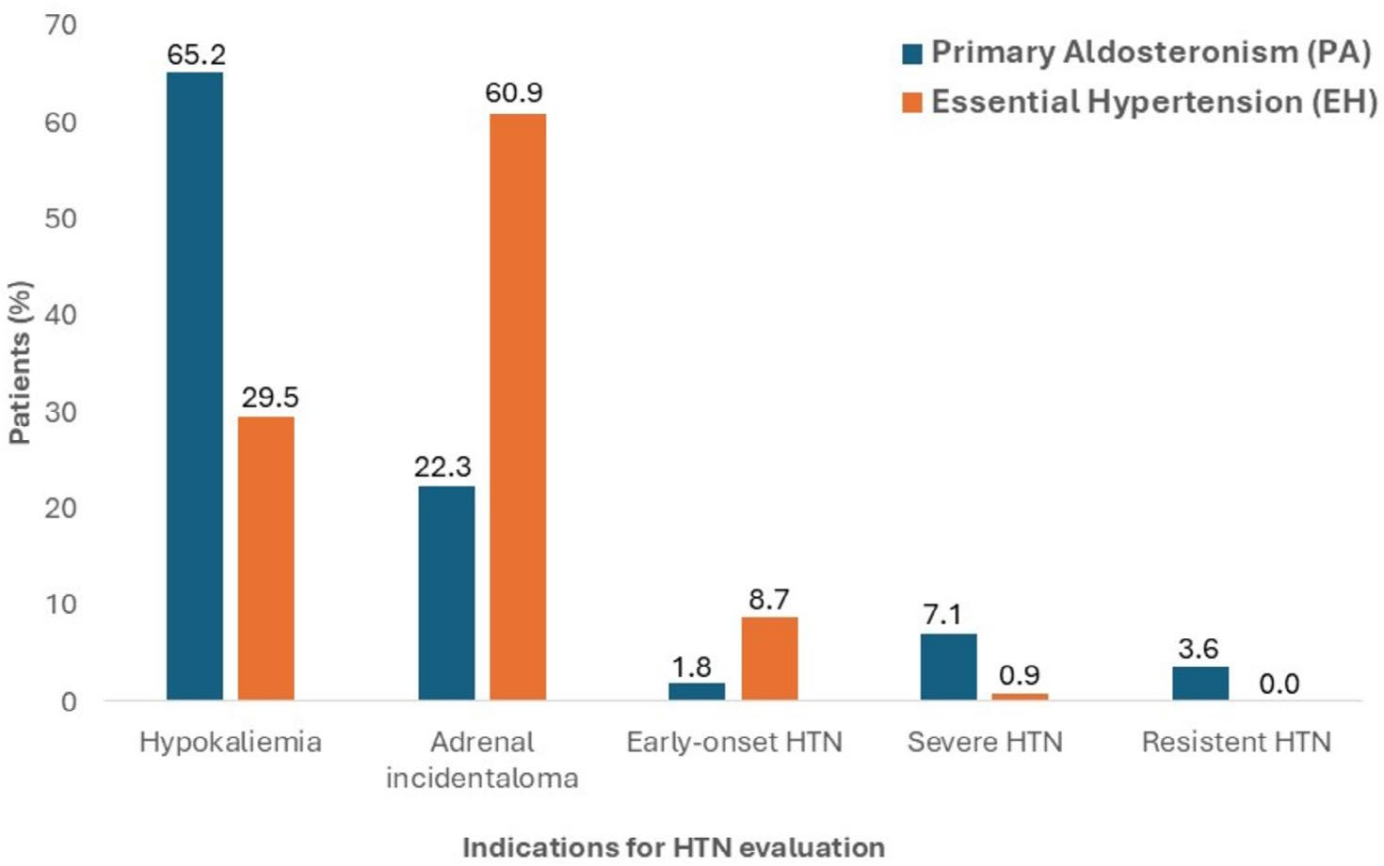
Indications for etiological evaluation of hypertension in patients with primary aldosteronism and essential hypertension. HTN: Hypertension.

Indications for etiological evaluation of hypertension in patients with PA and EH are shown in Figure 1. Hypokalemia was the leading cause for screening in the PA group (65.2%), while the evaluation of an adrenal incidentaloma was the most frequent indication in the EH group (60.9%) (*p* < 0.001). Patients with PA and those with EH were comparable regarding age, sex, smoking status, and history of type 2 diabetes. BMI was significantly higher in patients with EH (*p* = 0.04). Systolic and diastolic blood pressures were significantly higher in patients with PA (respectively *p* = 0.01 and p = 0.04). Potassium level was significantly lower in patients with PA (*p* < 0.01). PA was associated with unilateral adrenal adenoma in 48% of patients. The characteristics of the study population are summarized in Table 1.

**TABLE 1:** Characteristics of the study population.

	Primary aldosteronism (*N* = 112)	Essential hypertension (*N* = 115)	*p*
Age (years)	51 (44–57)	55 (37–65)	0.16
Male sex (%)	54.5%	52.2%	0.73
Smoking (%)	41.1%	40.2%	0.89
Type 2 diabetes (%)	26.8%	27.0%	0.98
Coronary disease (%)	2.7%	8.7%	0.05
Stroke (%)	9.9%	4.3%	0.10
Arrhythmia (%)	3.6%	0.9%	0.20
Lipid‐lowering therapy (%)	8.0%	18.3%	0.02
BMI (kg/m^2^)	29.7 (26.6–32.0)	30.8 (27.4–35.0)	0.04
Waist circumference (cm)			
Men	103.9 ± 13.7	105.9 ± 9.1	0.58
Women	99.8 ± 16.2	101.5 ± 11.8	0.72
SBP (mmHg)	150 (132–170)	140 (130–160)	0.01
DBP (mmHg)	90 (80–100)	80 (80–90)	0.04
Fasting blood glucose (mmol/L)			
Patients with diabetes	11.7 (6.7–13.3)	8.9 (6.1–11.1)	0.08
Patients without diabetes	5.3 (4.9–6.0)	5.2 (4.7–5.2)	0.38
HbA1c in patients with diabetes (%)	7.8 (7.1–10.7)	8.7 (6.7–10.3)	0.94
Potassium (mmol/L)	3.3 (3.0–3.6)	3.8 (3.5–4.2)	< 0.001
eGFR (mL/min/1.73 m^2^)	90.0 (75.0–102.0)	92.0 (78.0–105.0)	0.23
Baseline aldosterone (pg/mL)	251.5 (173.2–378.7)	90.2 (66.4–124.0)	< 0.001
ARR (pg/mL)/(pg/mL)	87.6 (48.7–165.5)	12.9 (7.5–17.7)	< 0.001

*Note:* Values are expressed as percentage (%), mean ± standard deviation, or median (25th–75th percentiles).

Abbreviations: ARR: aldosterone‐to‐renin Ratio; BMI: body mass index; DBP: diastolic blood pressure; eGFR: estimated glomerular filtration rate; SBP: systolic blood pressure.

Thirty subjects were on lipid‐lowering therapy, including 9 (8.0%) in the PA group and 21 (18.3%) in the EH group (*p* = 0.02). In patients not receiving lipid‐lowering therapy, triglyceride levels were significantly higher in the PA group (*p* = 0.04), whereas total cholesterol, HDL‐c, and LDL‐c did not differ significantly between groups (Table 2). In multivariable analyses adjusted for age and sex (Model 1), the association between PA and triglycerides was attenuated and no longer statistically significant (*B* = −0.16 [−0.38; 0.04], *p* = 0.12). Further adjustment for BMI and fasting plasma glucose (Model 2) resulted in additional attenuation of the associations, with no lipid parameter reaching statistical significance; only a borderline trend remained for triglycerides (*p* = 0.098) (Table 2). For total cholesterol, LDL‐c, and HDL‐c, the adjusted effect estimates (B coefficients) were negligible with broad confidence intervals encompassing zero, indicating that PA had no independent impact on these parameters in our cohort.

**TABLE 2:** Comparison of lipid parameters and multivariable regression analysis in patients with primary aldosteronism (PA) and controls with essential hypertension (EH) without lipid‐lowering treatment.

Lipid parameter (mmol/L)	PA (*n* = 103)	EH (*n* = 94)	*p*	Adjusted model 1^a^ B [95% CI]; *p*	Adjusted model 2^b^ B [95% CI]; *p*
Total cholesterol	4.66 ± 1.03	4.40 ± 1.03	0.23	−0.088 [−0.199; 0.024]; 0.124	−0.093 [−0.206; 0.019]; 0.104
Triglycerides	1.47 (1.13–1.92)	1.24 (1.02–1.71)	**0.04**	−0.168 [−0.382; 0.045]; 0.12	−0.182 [−0.397; 0.034]; 0.098
HDLc (Men)	0.96 (0.83–1.16)	0.96 (0.83–1.11)	0.95	0.044 [−0.038; 0.127]; 0.288	0.032 [−0.054; 0.118]; 0.459
HDLc (Women)	1.16 ± 0.28	1.16 ± 0.31	0.82	−0.005 [−0.060; 0.051]; 0.867	0.004 [−0.051; 0.060]; 0.875
LDLc	2.85 ± 0.88	2.77 ± 0.83	0.56	−0.051 [−0.148; 0.046]; 0.302	−0.059 [−0.157; 0.039]; 0.237

*Note:* Values are expressed as percentage (%), mean ± standard deviation, or median (25th–75th percentiles). Bold values indicate statistical significance (*p* < 0.05).

Abbreviations: B: unstandardized regression coefficient; CI: confidence Interval; HDLc: high‐density lipoprotein cholesterol; LDLc: low‐density lipoprotein cholesterol.

^a^ Adjusted model 1 (Total effect): Multivariable linear regression adjusted for age and sex. This model estimates the overall association between Primary Aldosteronism and lipid parameters.

^b^ Adjusted model 2 (Direct effect): Multivariable linear regression adjusted for age, sex, BMI, and fasting plasma glucose. This model assesses the direct association, considering BMI and glucose as potential mediators of aldosterone's metabolic effects.

**FIGURE 2:**
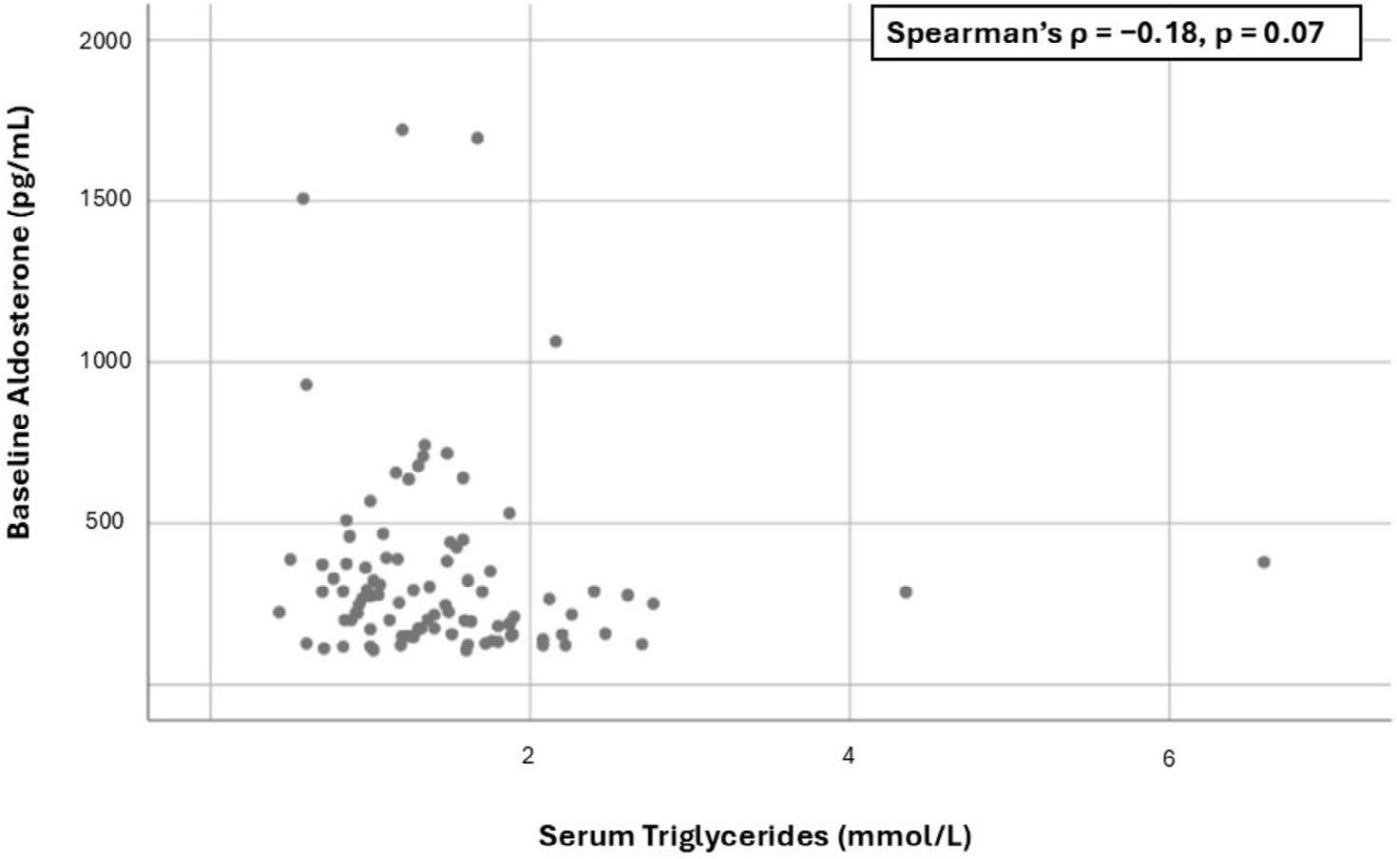
Correlation between baseline aldosterone levels and serum triglycerides in patients with primary aldosteronism.

No significant correlations were observed between lipid parameters and the biological severity of PA, as assessed by baseline aldosterone levels and the ARR in patients not receiving lipid‐lowering therapy (Table 3). An aldosterone level > 200 pg/mL was associated with lower triglyceride levels (1.35 (1.02–1.81) vs. 1.81 (1.35–2.15) mmol/L; *p* = 0.01). However, the overall continuous correlation between aldosterone and triglycerides was weak and did not reach statistical significance (Spearman's *ρ* = −0.18, *p* = 0.07) (Figure 2).

**TABLE 3:** Correlations of aldosterone levels and the aldosterone‐to‐renin ratio with lipid parameters in patients with primary aldosteronism without lipid‐lowering treatment (*n* = 103).

	Aldosterone		ARR	
	*ρ*	*p*	*ρ*	*p*
Total cholesterol	0.02	0.87	−0.02	0.86
Triglycerides	−0.18	0.07	−0.01	0.85
HDLc				
Men	0.28	0.15	0.12	0.12
Women	0.17	0.29	0.18	0.24
LDLc	−0.01	0.94	−0.06	0.57

Abbreviations: ARR: aldosterone‐to‐renin ratio; HDLc: high‐density lipoprotein cholesterol; LDLc: low‐density lipoprotein cholesterol; *ρ*: correlation coefficient.

**FIGURE 3:**
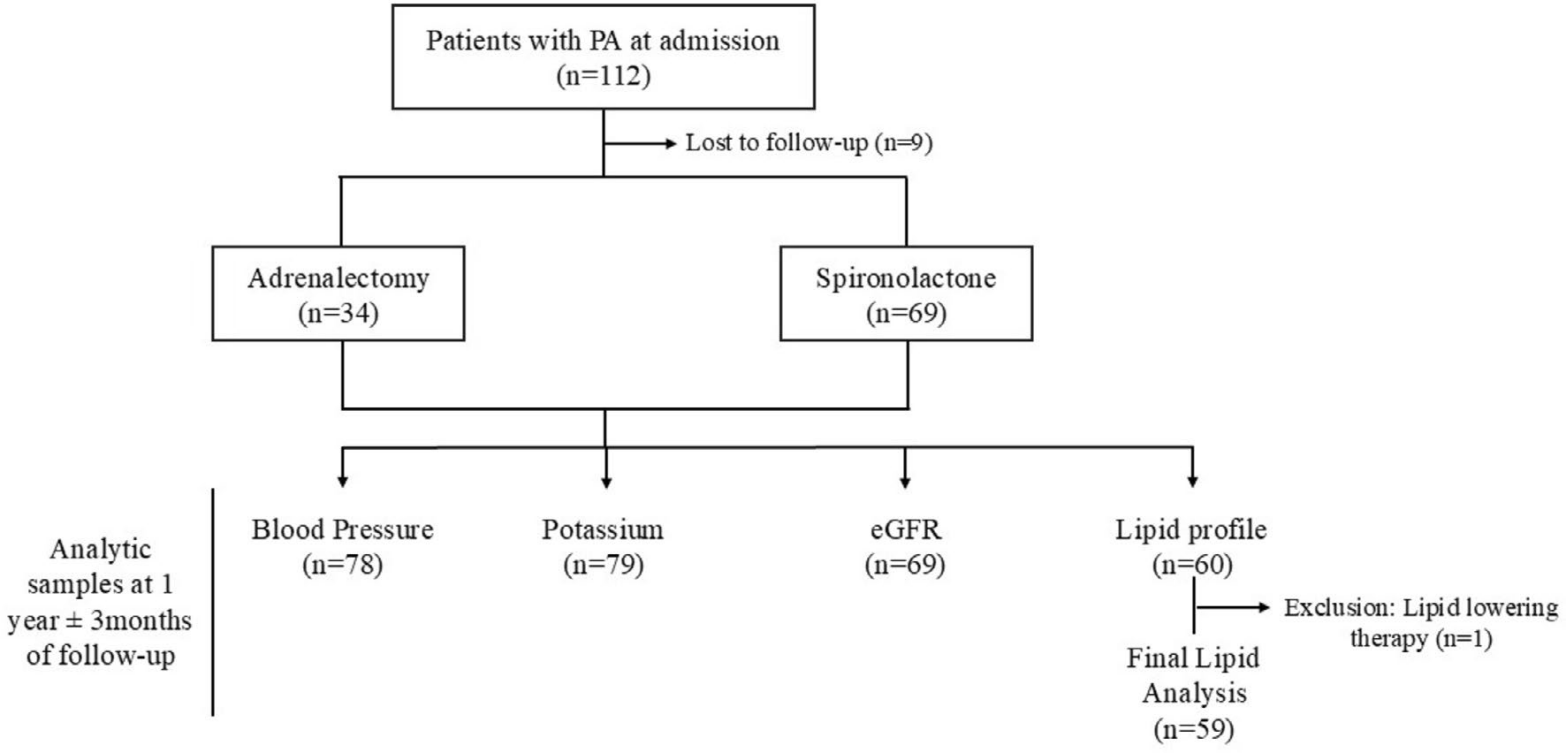
Flow diagram of the patients with primary aldosteronism.

Among the 112 patients with PA, 34 (30.4%) underwent surgical treatment (adrenalectomy), 69 (61.6%) received medical therapy with spironolactone, and 9 (8.0%) were lost to follow‐up. The derivation of the different analytic samples is illustrated in Figure 3. The median spironolactone dose in medically treated patients was 50 (50–100) mg/day. After 1 year of targeted therapy, lipid profiles were available for 60 patients: 59 who were not receiving lipid‐lowering treatment (10 who underwent adrenalectomy and 49 treated with spironolactone) and one patient on lipid‐lowering therapy. The latter was excluded from the analysis. A significant increase in triglyceride levels was observed among surgically treated patients (Table 4). Age, BMI, and baseline lipid parameters did not differ significantly between patients with and without follow‐up. In patients with follow‐up data, BMI was similar before and after specific treatment for PA (30.0 (26.9–32.1) vs. 30.7 (27.7–33.9) kg/m^2^; *p* = 0.25). Systolic and diastolic blood pressure decreased significantly, and serum potassium increased significantly after specific management of PA, whereas eGFR showed a significant decline (Table 5). The eGFR decline was observed in both surgically and medically treated patients but reached statistical significance only in the medically treated group (Table 5). No significant correlation was observed between changes in triglyceride levels and changes in eGFR following specific treatment (Spearman's *ρ* = −0.21, *p* = 0.16).

**TABLE 4:** Comparison of lipid parameters before and after specific treatment of primary aldosteronism in patients without lipid‐lowering treatment.

	Before specific treatment (*n* = 59)	After specific treatment (*n* = 59)	*p*
Total Cholesterol (mmol/L)	4.76 (3.83–5.33)	4.65 (4.16–5.33)	0.49
Adrenalectomy (*n* = 10)	3.98 (3.47–5.12)	4.68 (4.04–5.69)	0.09
Spironolactone (*n* = 49)	4.89 (3.93–5.33)	4.66 (4.14–5.17)	0.86
Triglycerides (mmol/L)	1.67 (1.23–2.05)	1.70 (1.16–2.42)	0.44
Adrenalectomy (*n* = 10)	1.53 (1.32–2.11)	2.08 (1.38–2.50)	**0.04**
Spironolactone (*n* = 49)	1.71 (1.19–1.99)	1.69 (1.12–2.18)	0.84
HDLc (mmol/L)	1.01 (0.83–1.22)	0.96 (0.85–1.24)	0.37
Adrenalectomy (*n* = 10)	0.96 (0.80–1.16)	0.91 (0.80–1.22)	0.49
Spironolactone (*n* = 49)	1.09 (0.91–1.32)	1.09 (0.85–1.37)	0.66
LDLc (mmol/L)	3.10 (2.30–3.41)	3.03 (2.25–3.52)	0.84
Adrenalectomy (*n* = 10)	3.10 (2.33–3.57)	3.52 (2.30–4.37)	0.41
Spironolactone (*n* = 49)	3.13 (2.30–3.39)	3.03 (2.22–3.44)	0.89

*Note:* Bold values indicate statistical significance (*p* < 0.05). Values are expressed as median (25th–75th percentiles).

Abbreviations: HDLc: high‐density lipoprotein cholesterol; LDLc: low‐density lipoprotein cholesterol.

**TABLE 5:** Comparison of blood pressure, serum potassium levels, and estimated glomerular filtration rate before and after specific treatment of primary aldosteronism.

	Before specific treatment (*n* = 78)	After specific treatment (*n* = 78)	*p*
SBP (mmHg)	150 (140–170)	130 (120–140)	< 0.01
Adrenalectomy (*n* = 25)	150 (135–180)	130 (120–140)	< 0.01
Spironolactone (*n* = 53)	150 (140–170)	130 (120–140)	< 0.01
DBP (mmHg)	90 (80–100)	80 (70–90)	< 0.01
Adrenalectomy (*n* = 25)	90 (80–90)	80 (70–90)	0.15
Spironolactone (*n* = 53)	80 (80–100)	80 (70–90)	0.04
Number of antihypertensive agents	2.0 (1.0–3.0)	2.0 (1.0–3.0)	0.80
Adrenalectomy (*n* = 25)	2.0 (1.0–4.0)	1.0 (0.0–2.0)	0.01
Spironolactone (*n* = 53)	2.0 (1.0–3.0)	2.0 (2.0–3.0)	0.11

	Before specific treatment (*n* = 79)	After specific treatment (*n* = 79)	*p*
Potassium (mmol/L)	3.3 (3.1–3.6)	4.2 (3.8–4.5)	< 0.01
Adrenalectomy (*n* = 24)	3.1 (2.9–3.4)	4.2 (3.9–4.5)	< 0.01
Spironolactone (*n* = 55)	3.4 (3.1–3.6)	4.2 (3.8–4.5)	< 0.01

	Before specific treatment (*n* = 69)	After specific treatment (*n* = 69)	*p*
eGFR (ml/min/1.73 m^2^)	84.9 ± 19.7	73.6 ± 24.9	< 0.01
Adrenalectomy (*n* = 23)	91.8 ± 21.4	85.2 ± 29.0	0.15
Spironolactone (*n* = 46)	81.5 ± 18.4	67.7 ± 20.9	< 0.01

*Note:* Values are expressed as percentage (%), mean ± standard deviation, or median (25th–75th percentiles).

Abbreviations: DBP: diastolic blood pressure; eGFR: estimated glomerular filtration rate; SBP: systolic blood pressure.

## 4 Discussion

The relationship between PA and lipid metabolism remains incompletely elucidated, and findings in the literature are inconsistent (Sun et al. 2024; Huang et al. 2021; Tsurutani et al. 2017; Matrozova et al. 2009; Bochud et al. 2006; Hannich et al. 2018; Jochmanová et al. 2013; Musani et al. 2013; Zhang et al. 2020; Adolf et al. 2016; Moon et al. 2021; Manosroi et al. 2022; Kaga et al. 2015). Data regarding North African cohorts remain scarce, leaving a geographical gap in the global understanding of PA‐related metabolic disorders. In this Tunisian cohort, patients with PA exhibited higher triglyceride levels than those with EH in unadjusted analyses. However, this association was attenuated after adjustment for age and sex and further weakened after additional adjustment for BMI and fasting glucose, with only a borderline trend remaining. This pattern suggests that the observed differences in triglycerides may be mediated by metabolic factors such as body composition and insulin resistance rather than a direct effect of aldosterone excess on lipid metabolism. A baseline aldosterone level > 200 pg/mL was associated with lower triglyceride levels (*p* = 0.01) and a trend towards a negative correlation (*ρ* = −018, *p* = 0.07) was observed between baseline aldosterone and triglyceride levels. Although the overall continuous association was weak and only borderline statistically significant, these findings raise the possibility of a favorable influence of aldosterone on triglyceride regulation. Consistent with this hypothesis, the longitudinal analysis demonstrated a significant increase in triglyceride levels among patients who underwent adrenalectomy, suggesting that preoperative aldosterone excess may have exerted a protective effect on triglyceride metabolism.

While several studies comparing patients with PA and EH found no significant differences between the groups (Sun et al. 2024; Huang et al. 2021; Matrozova et al. 2009), other investigations have reported a more favorable lipid profile in patients with PA (Sun et al. 2024; Zhang et al. 2020; Moon et al. 2021; Manosroi et al. 2022). In a recent meta‐analysis, Sun et al. (2024) found that triglyceride levels were significantly lower in subjects with PA. Manosroi et al. (2022) also observed lower triglyceride and LDL‐C levels in PA compared with EH patients, although both analyses reported substantial heterogeneity. Notably, and in line with our findings, the study of Moon el al. also identified an inverse association between aldosterone and triglyceride levels (Moon et al. 2021). Interestingly, patients with PA related to Conn's adenoma tend to display a more favorable lipid profile compared with those with bilateral adrenal hyperplasia, which may be attributed to the greater biological severity of aldosterone excess in Conn's adenoma (Musani et al. 2013; Zhu and Zhu 2023; Zhang et al. 2021; Somlóová et al. 2010).

Besides, some studies have shown that higher aldosterone levels were associated with a less favorable lipid and metabolic profile in the general population (Bochud et al. 2006; Hannich et al. 2018; Musani et al. 2013). Hannich et al. observed a positive correlation between plasma aldosterone concentration and both LDL‐C and non‐HDL‐C levels, along with a negative correlation with HDL‐C levels (Hannich et al. 2018). Similarly, Bochud et al. demonstrated that baseline aldosterone levels were independently associated with metabolic syndrome (Bochud et al. 2006). The authors attributed these findings to aldosterone‐induced stimulation of adipocytes, promoting lipid alterations (Hannich et al. 2018).

Consistent with our findings, two previous studies have also reported a worsening of the lipid profile following surgical treatment of PA (Adolf et al. 2016; Kaga et al. 2015), supporting the hypothesis that aldosterone excess may exert a beneficial effect on lipid metabolism. In a retrospective study of 215 patients with PA, Adolf et al. observed increased triglyceride levels and decreased HDL‐C concentrations after adrenalectomy or spironolactone therapy (Adolf et al. 2016). Similarly, Kaga et al. reported a deterioration in lipid parameters after adrenalectomy, with dyslipidemia developing in 18 of 39 patients who had normal preoperative lipid profiles (Kaga et al. 2015). In our cohort as well, triglyceride levels increased significantly after adrenalectomy. This phenomenon may be explained by a postoperative reduction in glomerular filtration rate secondary to the decline in aldosterone levels. Supporting this hypothesis, our study also demonstrated a significant decrease in eGFR following PA management. A reduction in lipoprotein lipase activity has been proposed as the most plausible explanation (Adolf et al. 2016; Moon et al. 2021; Kaga et al. 2015). However, we did not observe a significant association between individual changes in triglycerides and changes in eGFR. Therefore, the hypothesis that alterations in renal hemodynamics directly mediate lipid changes remains speculative and requires further investigation in larger longitudinal cohorts.

Alternative explanations, including changes in medical therapy, diet, weight, or lifestyle following diagnosis and surgical management, may also have contributed to the observed triglyceride increase. These factors were not systematically assessed and could partially explain the findings.

## 5 Strengths and Limitations of the Study

Our analysis adopted a multi‐faceted approach to evaluate the lipid profile in patients with PA. First, we conducted a cross‐sectional comparison of lipid parameters between patients with PA and a control group composed of hypertensive subjects in whom PA and other major causes of secondary hypertension were rigorously excluded. All participants were evaluated within the same hospital department and during the same period, ensuring consistency in assessment protocols and management. Next, we performed correlation analyses between lipid parameters and markers of PA biological severity. Finally, a longitudinal design was applied to assess changes in lipid parameters following specific therapeutic interventions and subsequent control of PA. In addition, the study provides data from a North African tertiary referral center, a population that is underrepresented in the current literature on PA and metabolic outcomes.

Our study nonetheless presents certain limitations. The EH group had a significantly higher BMI than the PA group. Although this difference may represent a potential confounder in the analysis of lipid metabolism, it likely reflects the inherent metabolic characteristics of EH, notably a greater predisposition to obesity. To mitigate this limitation, we performed a multivariable regression analysis adjusting for major confounding factors known to influence lipid metabolism, including age, sex, BMI, and fasting plasma glucose. In addition, our control group consisted of patients with EH who were hospitalized in a tertiary referral center. This group may represent a more severe or complex hypertensive phenotype than patients managed in primary care. However, this recruitment strategy ensured that all controls underwent the same rigorous diagnostic work‐up as the PA group, effectively ruling out PA and other secondary causes. Another limitation concerns the absence of subgroup analyses based on PA etiology (i.e., Conn's adenoma vs. bilateral adrenal hyperplasia). As these two conditions differ in their pathophysiological mechanisms and severity, they may exert distinct metabolic effects. Such an analysis was precluded by diagnostic uncertainty, as a definitive diagnosis was unavailable for many participants who lacked adrenal venous sampling data. Finally, the biochemical adequacy of medical treatment was assessed through clinical targets (blood pressure and potassium levels) rather than post‐treatment renin levels, consistent with clinical guidelines in effect during the study period, which may limit the assessment of complete mineralocorticoid receptor blockade.

## 6 Conclusion

In conclusion, our data show mixed associations between aldosterone and triglycerides. While the post‐treatment rise in triglycerides suggests a potential link, these findings require confirmation in prospective studies to better define the impact of PA on lipid metabolism.

## Author Contributions

Meriem Yazidi conceived and designed the study and wrote the first draft of the manuscript. Arige Abid carried out the research. Elyes Kammoun, Chayma Bel hadj Sliman, and Meriem Yazidi analyzed the data. All authors contributed to and approved the final draft of the manuscript.

## Ethics Statement

All procedures performed in studies involving human participants were in accordance with the ethical standards of the institutional and/or national research committee and with the 1964 Helsinki declaration and its later amendments or comparable ethical standards. This study was approved by the Ethics Committee of La Rabta University Hospital (Tunis, Tunisia).

## Consent

Informed consent was obtained from all individual participants included in the study.

## Conflicts of Interest

The authors declare no conflicts of interest.

## Data Availability

The data that support the findings of this study are available on request from the corresponding author. The data are not publicly available due to privacy or ethical restrictions.
